# Reliability and validity of the revised impact on family scale (RIOFS) in the hospital context

**DOI:** 10.1186/s41687-019-0118-1

**Published:** 2019-05-14

**Authors:** Yorschua F. Jalil, Gregory S. Villarroel, Alejandra A. Silva, Lilian S. Briceño, Vanessa Perez Ormeño, Nicolas S. Ibáñez, Paulina A. Méndez, Cristina F. Canales, Mireya A. Méndez

**Affiliations:** 1 Department of Kinesiology and Respiratory Rehabilitation, Hospital Josefina Martínez, Avenida Camilo Henríquez 3691, Puente Alto, Santiago, Chile; 2 Escuela de Kinesiología, Facultad de Ciencias de la Rehabilitación, Universidad Andrés Bello, Santiago, Chile; 3 Master of Science, Clinic Epidemiology, Universidad de la Frontera, Temuco, Chile; 4 School of Medicine, Pontificia Universidad Católica de Chile, Santiago, Chile; 5 Adjunct Instructor, Kinesiology Career, Health Sciences Department, Faculty of Medicine, Pontificia Universidad Católica de Chile, Santiago, Chile

**Keywords:** Chronic illness, Family burden, Care givers, Impact on family scale, Children, Reliability, Validity

## Abstract

**Background:**

The lack of formal instruments to measure Burden in primary caregivers of Children in a hospital context is limited because mostly of published instruments are related to cancer survivors, ambulatory environment or general context for children with chronic conditions, but none of them adapted property to prolonged hospitalization context. This leaves the rising population of hospitalized chronic children’s caregivers without a proper assessment. The aim of this study was to develop a version of the Revised Impact on Family Scale adapted to primary caregivers of chronic hospitalized children. A cross-sectional study with two main stages was conducted. The first one describes the linguistic and contextual adaptation process of the instrument, and the second refers to the psychometric testing and analysis..

**Results:**

Less than 15% of the participants expressed problems with some adapted items in the scale. Eighty-six caregivers were evaluated at Josefina Martinez Hospital, mostly female (34.2 ± 11.6 years old). Majority of participants were graduated from high school, salaried employee and mothers of the chronic child. The scale exhibits a high level of internal consistency (Cronbach’s alpha 0.73), excellent intra-observer reliability (Intraclass Correlation Coefficient 0.9), acceptable empirical evaluation of content validity and low and negative construct validity (Pearson’s correlation coefficient − 0.23).

**Conclusions:**

This adapted version of the Revised Impact on Family Scale to the hospital context is a reliable, valid, self-administered and simple instrument to implement in order to assess the burden of primary caregivers with chronic hospitalized children.

## Background

In the last two decades, survival of severely ill children has increased due to technological advances and new health strategies changing the epidemiology of childhood diseases and guiding them towards chronicity [1–3]. However, prognosis, life expectancy, health related quality of life (HRQoL) and quality of life (QoL) can be affected [4]. Functional limitations and long-time hospitalization also can affect their family environment since caregivers usually bear important and multidimensional responsibilities over the time [4, 5]. The effective management of these problems associated with caregiving is a main challenge for involved parents, affecting their physical and psychological health, becoming a threat for whole family function [6–8].

Burden and family impact measures, associated to different scenarios of the chronic child care, are key aspects to achieve a proper management of this population. Inadequate assessment of the situation and non-systematic interventions from the health care providers can leave families to struggle alone in order to manage a wide child’s health issues during a hospitalization [8, 9]. Early detection of underlying problems can help to offer timely and appropriate health support to prevent family malfunction during child’s prolonged hospitalization [9, 10]. Learn to face, for example, how the transition from a long stay hospital to the child’s home can impact the family environment, which nowadays is part of their gold standard management [4, 9, 11, 12]..

Sadly, instruments to measure caregiver burden in different contexts are not always available. Factors such as translation difficulties, cultural differences between populations, absence of a real family environment and context of application may explain the lack of formal instruments. For instance, different clinical scenarios of patient care, e.g. homecare, intensive care unit or a long-term rehabilitation facility, might affect the applicability of current scales mainly designed for the ambulatory context [13, 14]. Indeed, there is few formal adapted instruments for burden assessment in an hospital context that can be compared later in other context such as home [15]. Mostly of published instruments for caregiver’s burden are related to cancer survivors, local environments or general context of children with chronic conditions, but none of them adapted property to chronic hospital context [6, 9, 16, 17]..

The Impact On Family Scale (IOFS) [18–20] is an instrument originally designed to assess family burden in the pediatric ambulatory care context. The initial version of it consisted in 27 items grouped in four factors (financial, familial/social, personal strain and mastery), however, a “Revised version of the IOFS” (RIOFS-Table 1) considering only 15 items has shown better psychometric properties than the original IOFS, recommending its use as a shorter, representative and more reliable single factor solution scale [7, 21].

**Table 1:** English version of the RIOFS

1	Our family gives up things because of my child’s illness.
2	People in the neighborhood treat us specially because of my child’s illness
3	We see family and friends less because of the illness
4	I don’t have much time left over for other family members after caring for my child.
5	We have little desire to go out because of my child’s illness.
6	Because of the illness, we are not able to travel out of the city.
7	Sometimes we have to change plans about going out at the last minute because of my child’s state.
8	Sometimes I wonder whether my child should be treated “specially” or the same as a normal child.
9	I think about not having more children because of the illness.
10	Nobody understands the burden I carry.
11	Traveling to the hospital is a strain on me.
12	Sometimes I feel like we live on a roller coaster: in crisis when my child is acutely ill. OK when things are stable.
13	It is hard to find a reliable person to take care of my child.
14	I live from day to day and don’t plan for the future.
15	Fatigue is a problem for me because of my child’s illness.

For each item an answer of four choices should be considered. “1. Strongly agree; 2. Agree; 3 Disagree; 4. Strongly disagree”

Different cross-culturally adaptions of the IOFS and RIOFS have been developed, including versions in Turkey, Germany, Brazil and France [22, 23]. These adaptations have evidenced the existence of cultural and linguistic differences leading to erroneous interpretations without the formal adaptation process [24]. The performance of either IOFS or RIOFS in the hospital context remains uncertain, leaving the chronic hospitalized children’s caregiver without a formal burden assessment [7, 23]. The purpose of this study was to develop a formal version of the Revised Impact on Family Scale adapted for primary caregivers in the hospital context of chronically ill children.

## Methods

### Study design

We conducted a descriptive cross-sectional study for contextual and linguistic adaptation of an instrument for caregiver burden assessment. The entire process was divided in two stages: First, a five step linguistic and contextual instrument adaptation was performed [25–27]. Second, a psychometric analysis of the adapted instrument obtained in the first stage was conducted [26]..

#### Cross-cultural linguistic and contextual adaptation

**Fig. 1:**
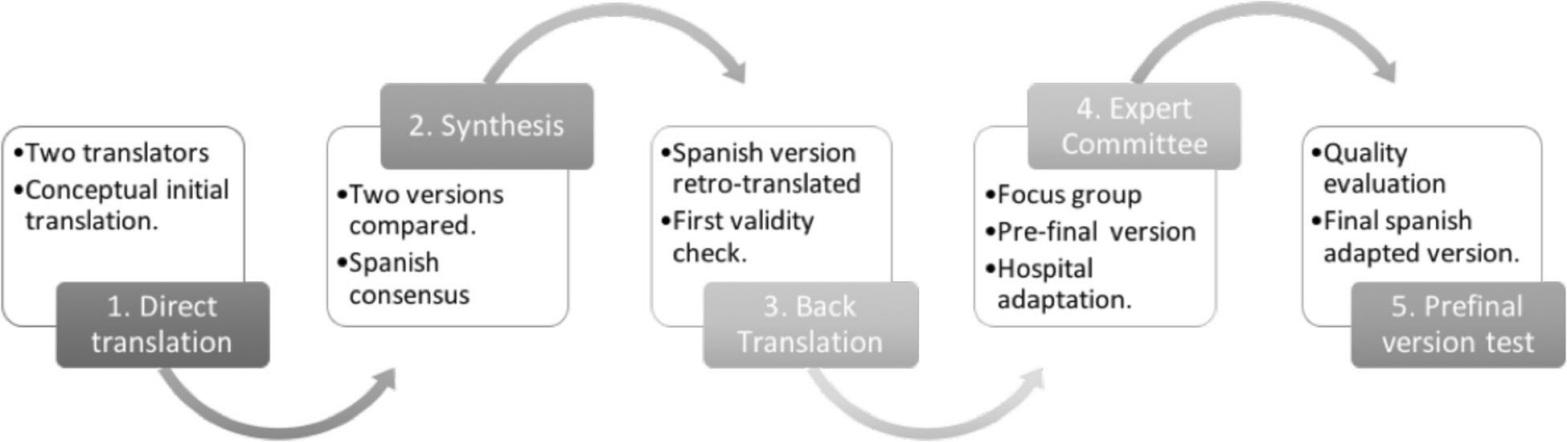
Cross-cultural linguistic and contextual adaptation process

This process considered five steps (Fig. 1):

1. Direct translation: Conceptual initial translation was made by two bilingual translators; whose native language was Spanish. The first translator knew the objectives of the study and the features which the scale intended to measure and has previous experience in technical text reduction. The second translator did not have any previous knowledge about the scale nor the study aims, offering a translation adjusted to popular language. This translator also highlights ambiguous meaning in the original questionnaire, detecting comprehension difficulties derived from the use of technical words.
2. Synthesis of the translations: The two translators, plus a recording observer, compared and synthesized both versions of the translated scale. They identified and discussed the differences between reports of the translation process. A Spanish consensus version of the RIOFS emerged from this step.
3. Back Translation: At this stage, the Spanish consensus version was translated into English by two different bilingual professional translators, whose native language was English (same as original language questionnaire). Difficult wording and conceptual errors in the back translation were highlighted in order to accomplish a first validity check.
4. Expert Committee: A multidisciplinary committee of experts (1 methodologist, 1 pneumologist specialized in chronic respiratory diseases, 1 psychologist, 1 social worker, 2 physiotherapists and 2 registered nurses) developed a single consolidated pre-final scale from the synthesis version in Spanish (after translation process, once revised and compared with the back translation). This version met the requirements to be comprehensible by a 12 years old person (evaluated by linguist and the expert committee). The committee also evaluated the applicability to the hospital context of all items, using a focus group strategy they elaborated the adapted version of the RIOFS. Every item was analyzed and adapted to the hospital context considering previous reports of translation process (provided by translators) and discussion between all committee members. This focus group was moderated by the main author, who registered the comments and prepared several reports to discriminate key information in order to achieve consensus. The ultimate result from this stage was the pre-final Spanish version of the RIOFS adapted to the hospital context prior to field test. This version, as the original RIOFS, consider 15 items with an agree-based response from 1 to 4 (Strongly agree to Strongly disagree)
5. Test of the prefinal version: This step was necessary to evaluate the quality of the pre-final consolidated version adapted to the hospital context. Thirty-six caregivers from the Hospital Josefina Martinez (HJM), Santiago, Chile; were recruited by 4 members of the research team, also part of the clinical staff, who extend them the informed consent and explain the main idea of this step. Caregivers must answered the new version and identified possible difficulties in understanding questionnaire instructions, answer options and/or items themselves. An appropriate pre-final version was considered when 15% or less of the participants, according to Ramada recommendations, reported any difficulties to answer the questionnaire [26].

#### Psychometric testing and analysis of the adapted instrument

Psychometrics properties, including validity and reliability, of the instrument obtained from the first stage were analyzed.

Validity analysis: Content and construct validity was considered. Content was based on an empirical evaluation by the expert committee. Construct validity was determined by its convergent variant. The application of a similar construct scale, the Family Apgar scale (currently used as part of the family impact measurement tools in the periodic evaluation at home), was performed simultaneously whit the adapted RIOFS [19, 28].

Reliability analysis: Internal consistency and intra-observer reliability were evaluated. For the internal consistency, all caregivers were considered, including those ones evaluated in the pre-test stage. For the intra-observer reliability, a second measurement was necessary to the same caregivers after 2–3 weeks from the initial one. This temporary margin was established to avoid memory bias (less than 2 weeks), and to avoid that the object of measurement changed significantly between assessments (no more than 3 weeks).

### Participants

Ethical approval was obtained from the South East Scientific Ethics Committee of Metropolitan Area (Santiago, Chile), and informed consent was signed by every caregiver who completed the survey prior to beginning the study.

All caregivers of inpatient children at Hospital Josefina Martinez between November 2016 and June 2017 were considered according to the following inclusion criteria: 1. To be a caregiver of a chronic inpatient child (defined according to pre-stablish criteria provided by social work team, such as visits, legal custody and care offer to the child); and 2. Level of education that allowed them to read and write (elementary school stablished by social worker and caregiver interview). Caregivers with some degree of cognitive impairment and those with a child undergoing an acute exacerbation were excluded.

Sample size was estimated according to Tinsley et al. criteria. The latter is based on variability maximization of answers, which proposes 5 to 10 subjects per item [29]. Considering that RIOFS integrates 15 items, a minimum of 75 caregivers were required (no item was dropped or added in the first stage of the scale adaptation).

### Data analysis

Mean, standard deviation, frequencies and percentages were used for sample description as appropriate. Both statistical programs Stata 12.0 and SPSS 22.0 were used for the complete analysis.

#### For the reliability study

Cronbach’s alpha coefficient was calculated as the measurement of internal consistency. Intra-observer reliability was analyzed through the intraclass correlation coefficient (ICC). Global and inter-item relation was considered using Kappa index agreement.

#### For the validity study

An empirical evaluation based on the interdisciplinary expert’s committee debate (during the process described above) was considered for content validity. For construct validity, its “convergent” branch was used, which consisted in examining the correlation between the modified RIOFS and the Apgar Familiar Scale (FA).

## Results

### Linguistic, cultural and hospital adaptation process

The first stage result is the Spanish hospital adapted version of the RIOFS (Table 2). This final implemented version was achieved after the adjustments of some items in order to make them applicable to our context. Item number 12 presented semantic and “cultural” problems in its conception, since “roller coaster” is a common term used in the United States to express the presence of ups and downs. For our context was necessary to adapt it using the linguistic technical resource of “explicitation”, which consists of adding an explanation following the desired word (a roller coaster, with highs and lows). It was necessary to understand that it is about the highs and lows in the health status of the child, requiring a more detailed description such as “Sometimes I feel as if we were living on a roller coaster (with highs and lows): in crisis when my child is unbalanced and well when he is stable”. The item number 13 originally states “It is difficult to find someone you trust to take care of my child”. This item did not apply to the hospital context since caregivers do not need to find someone to take care of their child (inpatient child). It was adapted to “It is difficult to place my trust in the people who take care of my child”.

**Table 2:** Spanish hospital adapted version of the RIOFS

1	Nuestra familia deja de hacer cosas debido a la enfermedad de mi hijo/a.
2	La gente del vecindario nos trata de manera diferente o no deseada debido a la enfermedad de mi hijo/a.
3	Vemos menos a nuestros familiares y amigos debido a la enfermedad mi hijo/a.
4	No me queda mucho tiempo para otros miembros de mi familia después de cuidar a mi hijo(a).
5	Tenemos pocas ganas de salir debido a la enfermedad de mi hijo/a.
6	Debido a la enfermedad mi hijo/a no podemos viajar fuera de la ciudad.
7	A veces tenemos que cambiar los planes de salir, a último minuto, debido al agravamiento del estado de salud de mi hijo/a.
8	A veces me pregunto si mi hijo/a debería ser tratado de manera “especial” o igual que a otro niño de su edad.
9	Pienso en no tener más hijos debido a la enfermedad de mi hijo/a.
10	Nadie entiende la carga que significa la enfermedad de mi hijo/a para mí
11	Ir al hospital es un estrés para mí.
12	A veces siento como si viviéramos en una montaña rusa (con altos y bajos): en crisis cuando mi hijo(a) está descompensado (a) y bien cuando está estable.
13	Es difícil depositar mi confianza en las personas que cuidan a mi hijo/a.
14	Vivo el día a día y no hago planes para el futuro.
15	No me puedo permitir estar cansada/o por la enfermedad de mi hijo/a.

For each item an answer of four choices should be considered. “1. Muy de acuerdo; 2. De acuerdo; 3 Desacuerdo; 4. Muy en desacuerdo”

Pre-final version (pre-test stage) and final version were the same since only 11.1% (4 observations) of the caregivers reported some difficulties in the evaluation process. The collected comments reflected some degree of conflict in 3 specific items (8–10, and). Item 8 registered 2 apprehensions, corresponding to 5.6% of the pre-test sample. This item showed that 2 answers are possible (“my child should be treated specially” or “the same as a normal child”). Item 9 corresponds to “I think about not having more children because of the illness” registered 1 apprehension. In this one it realizes that the real reason for this kind of decision was not the child illness, but family planning. Item 10 corresponding to “Nobody understands the burden I carry” also registered 1 observation, directly related to the understanding of the item, highlighting that the child in question is not the source of their sorrows, it is the disease that they suffer from.

### Socio-demographic characteristics

**Fig. 2:**
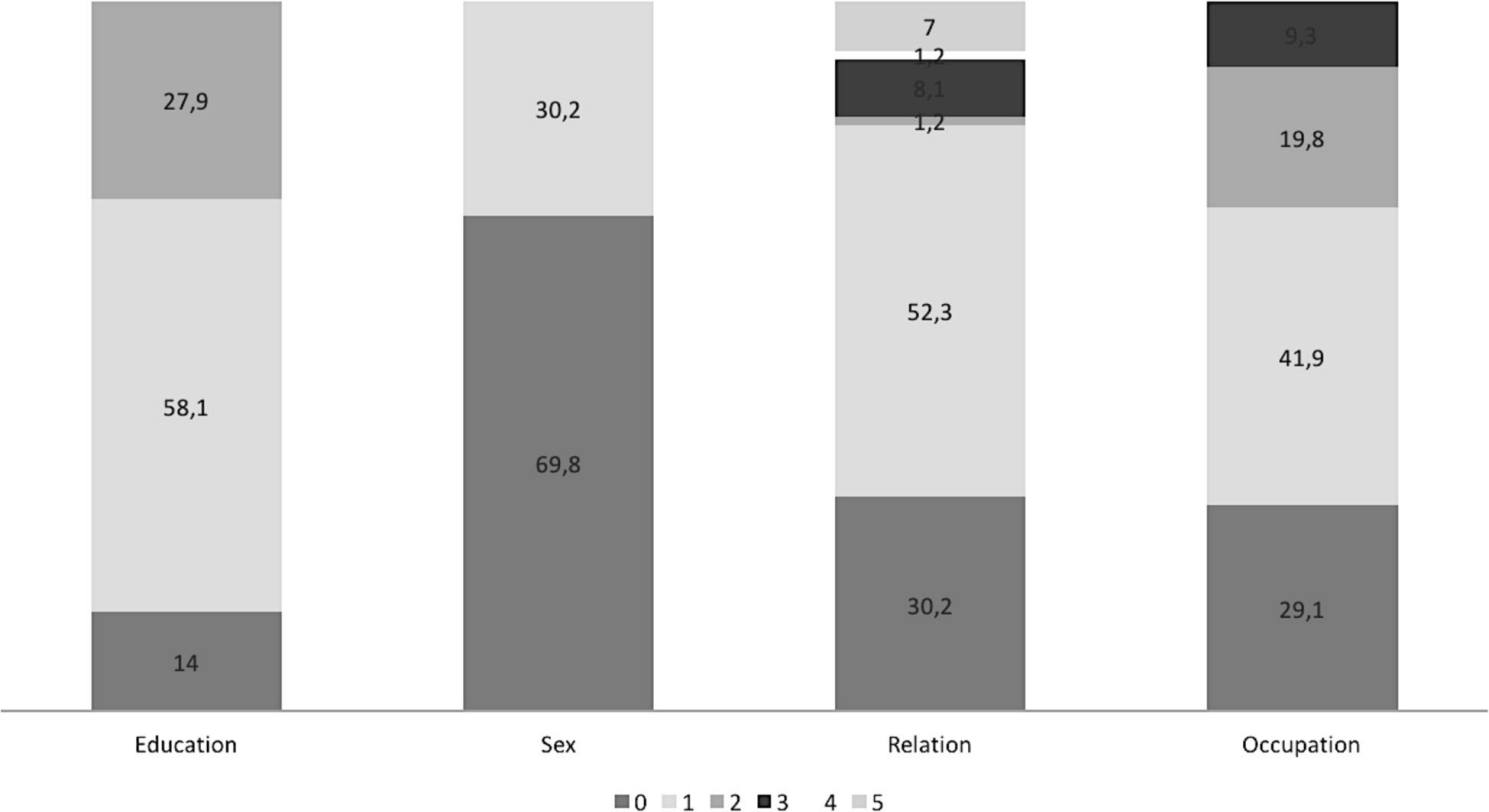
Socio-demographics characteristics. For Education Level: Primary (0), Secondary (1) and Higher Education (2). For Sex: Female (0) and Male (1). For Relation: Father (0), Mother (1), Siblings (2), Grandparents (3), Uncle/Aunt (4) and non-family (5). For Occupation: Housewives (0), Dependent Worker (1), Independent Worker (2) and Unemployed (3)

Eighty-six caregivers (including 36 evaluated in the pretest stage) were considered for the final analysis. Among them, 60 (69.8%) of the respondents were female, with a mean ± SD age of 34.2 ± 11.6 years, slightly higher than men (30.4 ± 7.4 years). Fifty caregivers (58.1%) have completed high school, where 52.3% of them correspond to mothers of chronically inpatient children. The second proportion of caregivers corresponds to fathers (30.2%), so parents are the main caregivers (82.5%). Sixty-two percent declared had an employment, where 41.9% are salaried employee and the rest are freelance. The other declared occupation was housewife (29.1%) (Fig. 2).

### Reliability

Good internal consistency was found with a Cronbach’s alpha coefficient value of 0.73. Analysis of the intra-observer was differentiated into 2 parts. We used the total score of the adapted instrument, obtaining an excellent ICC (0.9). The second part considered the intra-observer reliability calculation between the different items ranging from 0.62 to 0.83 (Table 3).

**Table 3:** Intra-observer reliability calculation

Item	Kappa value	SD	*P* value
1	0,734	0,059	0,000
2	0,667	0,076	0,000
3	0,668	0,066	0,000
4	0,670	0,065	0,000
5	0,696	0,061	0,000
6	0,793	0,055	0,000
7	0,714	0,063	0,000
8	0,673	0,062	0,000
9	0,621	0,065	0,000
10	0,790	0,053	0,000
11	0,684	0,067	0,000
12	0,731	0,060	0,000
13	0,643	0,064	0,000
14	0,776	0,056	0,000
15	0,826	0,049	0,000

### Validity

From the clinicians and expert perspective, the committee assessed the scale as a whole, declaring it representative of “Family burden/impact” construct in hospitalized chronic children caregivers, reporting acceptable content validity. Similar assumption could be made from the caregiver’s perspective, since in the pre-test stage, none of them declared not covered aspects of this domain in the questionnaire. Construct validity found a low and negative correlation with a Pearson value of − 0.25 for the initial evaluation and − 0.23 for the final measurement.

### Discussion

The adapted version of the RIOFS in the hospital context has been presented as a simple answering tool, self-administered and adjusted to the required context. Despite reported difficulties in the pre-test stage, they were less than the expected proportion (15%) and corresponded mostly to observations of a comprehensive nature and in a lesser proportion to interpretative difficulties, where susceptibilities inherent to the treatment of ill children prevailed. Words such as “burden” and “special treatment” seem to be key in the origin of these difficulties, however, they are caregiver-dependent and are expected in some way, considering that sensitive topics such as personal and family function are the main topics of interest.

The proposed scale exhibits “acceptable” psychometric properties of reliability and validity that would validate its application in chronic inpatient children caregiver’s population. These results could be explained by the fact that RIOFS is a one-factor solution, unlike the original IOFS which exhibit poorer psychometric properties than the revised version. This does not guarantee that these properties were maintained after the translation and adaptation process. Our results suggest that they remained acceptable after this process. The internal consistency showed “acceptable” values (0.73 Cronbach’s alpha), framed in the category of “good” (values> 0.7), as reported by Stein et al. in other experiences with outpatient populations, exhibiting values ​​of Cronbach’s alpha of 0.89, 0.87 and 0.83. These values ​​do not differ from our findings [7]. The difference could be explained by two factors, the first by the context where it applies, there are no previous experiences in hospitalized children. The second determining factor is the sample size, which is similar to that used in the Turkish adaptation (85 patients), but lesser than other adaptations (over 200 patients) [23, 28, 30, 31]. Although it is true that the minimum standard of patients is met to perform the psychometric adaptation, the differences in internal consistency could be explained by the greater variability associated with the smaller sample size. It should be considered that given the characteristics of the HJM, a long-term hospital (mean of 1.2 years per child admitted), with an average of only 10 admissions per year.

For intra-observer reliability, the 0.9 value found for ICC was considered excellent, very close to the 0.87 ICC reported by Boudas et al. in the French adaptation. This is the closest comparable adaptation experience of the RIOFS, since the other reports of cultural adaptation were made using the original IOFS [26]. Even if we decided to compare these last adaptations with the ICC value found in this study, we would find that they are very similar: 0.9 and 0.94 for the Brazilian and Turkish versions respectively [27].

The intra-observer reliability evaluated for each item separately through the Kappa coefficient impresses being “moderate to strong” with a range from 0.621 to 0.826. This agrees with the intra-observer reliability for the complete scale. The items with highest reliability are those related to factors non-modifiable at short term, such as “ Fatigue is a problem for me because of my child’s illness” (item 15) or “no one understands the burden I carry” (item 10). Low reliability items seems to be related to daily health care, as item 13 “It is difficult to find a reliable person to take care of my child”, statement that could be impacted (positively or negatively) by single action of the health team during any day..

It is necessary to mention that since it is a self-administered scale, intra-observer reliability seems an inherent property. However, different factors such as caregiver’s reception to the observer initial instruction could change according to observer attitude or caregiver perception of it, being an important issue assess this influence through the intra-observer reliability analysis.

Regarding the convergent construct validity, that was categorized as low and negative, it seems that the FA scale points to a similar, but not equivalent construct, in contrast with the RIOFS. The FA, widely used as part of the QoL measurements by Chilean state home children care programs, shows how family members perceive the level of functioning of the family unit globally, while the RIOFS aims to determine the impact or burden that the chronic child disease generates on the family. This affects its comparability, explaining its low and negative correlation. The latter exhibits a behavior more likely expected for construct validity in its concurrent variant, which is when scales that points to different constructs are compared. Thus, if consider the application of FA as a measure of concurrent construct validity, the appropriate psychometric behavior of the proposed instrument is reinforced.

### Limitations

This study has several limitations. First of them is related to the expected ability of the caregivers to write, read and to comprehend the questions in the scale. The second limitation is the sample size, which could be considered small in comparation to major studies. The study was also conducted in a single institution; however, considering the arguments in the discussion section, we don’t expect that these factors alters significantly our findings.

## Conclusion

In the present study the Spanish version of the RIOFS adapted to the hospital context is a reliable, valid, self-administered and simple instrument to implement in order to assess the impact/burden that diseases of chronically hospitalized children have on their caregivers and family environment. Further studies are needed in order to integrate more caregivers, allowing characterize the perceptions of this population about different clinical scenarios and interventions in health care..

## Abbreviations

FA: Family Apgar
ICC: Intraclass correlation coefficient
IOFS: Impact On Family Scale
QoL: Quality of Life
RIOFS: Revised version of the IOFS
